# Factors Influencing the Use of G-CSF in Drug-Induced Agranulocytosis

**DOI:** 10.3390/hematolrep18010014

**Published:** 2026-02-03

**Authors:** Emmanuel Andrès, Jean-Edouard Terrade, Xavier Jannot, Noel Lorenzo-Villalba

**Affiliations:** Service de Médecine Interne, Hôpital de Hautepierre, Hôpitaux Universitaires de Strasbourg, 67000 Strasbourg, France; jean-edouard.terrade@chru-strasbourg.fr (J.-E.T.); xavier.jannot@chru-strasbourg.fr (X.J.); noel.lorenzo-villalba@chru-strasbourg.fr (N.L.-V.)

**Keywords:** drug-induced agranulocytosis, granulocyte-colony stimulating factor (G-CSF), neutropenia, hematologic toxicity, supportive care, pharmacogenetics, adverse drug reaction, risk-adapted therapy

## Abstract

Drug-induced agranulocytosis is a rare but life-threatening adverse reaction associated with numerous non-chemotherapy drugs. Management relies on immediate drug withdrawal, infection control, and, in selected patients, administration of granulocyte-colony stimulating factor (G-CSF). This review summarizes current knowledge on the determinants of epidemiology, clinical presentation, hematologic and biologic features, comorbidities, and outcomes influencing the decision to introduce G-CSF in drug-induced agranulocytosis. Evidence from observational studies and meta-analyses suggests that G-CSF shortens neutropenia duration and hospitalization, although its impact on mortality remains uncertain. The decision to use G-CSF should consider initial neutrophil count, presence of severe infection or sepsis, age, and comorbidities. Despite the accumulated experience, randomized controlled trials are still lacking, and treatment algorithms remain empirical.

## 1. Introduction

Drug-induced agranulocytosis (DIA) is an uncommon but potentially life-threatening idiosyncratic reaction. Severe neutropenia is defined by an absolute neutrophil count (ANC) of less than 0.5 × 10^9^/L and agranulocytosis is defined by an ANC of less than 0.2 × 10^9^/L. DIA is frequently accompanied by fever, sepsis, or oropharyngeal ulcerations. It arises following exposure to a broad spectrum of non-cytotoxic agents, including antithyroid medications, antibiotics (e.g., β-lactams, sulfonamides), antipsychotics (notably clozapine), metamizole and certain analgesics and antiepileptics. Despite its rarity, estimated at 1–5 cases per million persons annually, DIA remains associated with considerable morbidity and a mortality rate of 5–20%, particularly in elderly or septic patients [1,2]. The introduction of granulocyte colony-stimulating factor (G-CSF) in the 1990s revolutionized the management of chemotherapy-induced neutropenia by accelerating myeloid recovery and reducing infectious complications. The activation of G-CSF-R induces a downstream signaling cascades that include the Janus Kinase, the Phosphoinositide-3-kinase/Protein kinase B, signal transducers activators of transcription (STAT) and mitogen-activated protein kinase pathways. The transcription factors of STAT are positively activated by the interaction while suppressors of cytokine signaling proteins act as negative regulators of G-CSF-R, resulting in maintaining an optimal expression of the receptor. These signaling cascades are necessary for the proliferation, differentiation, and survival of blood cells from the neutrophilic granulocytic lineage [3]. Its off-label use in non-chemotherapy DIA soon followed, though evidence supporting improved survival remains inconclusive [4,5]. Observational data suggest that G-CSF may shorten the duration of neutropenia and hospital stay, yet the magnitude of clinical benefit and optimal dosing remain debated [6]. This review aims to discuss current evidence regarding epidemiologic, clinical, hematologic, and biologic determinants of outcome in DIA, while reassessing the therapeutic role of G-CSF in this heterogeneous disorder. Particular attention is given to age, comorbidities, infection severity, and drug class, as well as emerging insights into genetic susceptibility and immune-mediated mechanisms that may inform precision management strategies.

## 2. Epidemiological Determinants

The epidemiology of drug-induced agranulocytosis (DIA) varies across regions and is strongly influenced by prescribing patterns (Table 1). Large pharmacovigilance databases and national registries consistently implicate antibiotics (particularly β-lactams and cotrimoxazole), antithyroid drugs (methimazole, propylthiouracil), antipsychotics (notably clozapine), and nonsteroidal anti-inflammatory drugs (NSAIDs) [7,8]. Geographic differences reflect drug availability and monitoring policies; for instance, metamizole-related agranulocytosis remains prevalent in parts of Europe and Latin America but rare in regions where the drug is restricted.

The median age at onset is typically between 50 and 60 years, with a modest female predominance, likely reflecting differential exposure to antithyroid and antibiotic therapies. The latency period between drug initiation and agranulocytosis usually ranges from 2 to 12 weeks, though earlier or delayed presentations may occur depending on cumulative dose and immune sensitization [9].

Recent studies highlight the contribution of genetic susceptibility and immune-mediated mechanisms. Specific HLA haplotypes, including HLA-B38 and DRB1*04:02, have been associated with clozapine- and methimazole-induced agranulocytosis, suggesting a shared immunogenetic basis [10]. Environmental cofactors, such as concurrent infections or metabolic polymorphisms affecting drug detoxification, may further modulate the risk.

Despite improved diagnostic and therapeutic strategies, mortality remains between 5% and 10% in contemporary series [11], predominantly affecting elderly or septic patients. Adverse outcomes are closely linked to delayed recognition, profound neutropenia duration, and comorbidities such as renal or hepatic impairment.

## 3. Clinical and Hematologic Determinants

Clinical manifestations of (DIA) are largely dominated by infection-related symptoms, which often develop abruptly. The most frequent presentations include fever, sore throat, oral or gingival ulcerations, and pneumonia, frequently progressing to severe sepsis or septic shock. Bacteremia occurs in up to 60% of cases, predominantly due to Gram-negative bacilli (notably *Escherichia coli* and *Pseudomonas aeruginosa*) or *Staphylococcus aureus* [12]. Oropharyngeal and gastrointestinal mucosal breaches serve as major portals of entry for these pathogens.

Hematologically, a complete blood count typically reveals isolated neutropenia with preserved erythroid and megakaryocytic lineages in 45% of patients. Anaemia occurs in approximately 50% of patients, and thrombocytopenia in 25%. In our experience, pancytopenia affects about 5–10% of patients [13]. Bone marrow aspiration or biopsy usually shows maturation arrest or marked depletion of granulocytic precursors, consistent with immune-mediated destruction or toxic suppression of myelopoiesis.

The depth and duration of neutropenia are the principal prognostic determinants. Patients with ANC < 0.1 × 10^9^/L face the highest risk of invasive infection and mortality. Early identification and rapid institution of broad-spectrum antimicrobial therapy are therefore critical. Elevated C-reactive protein (CRP) and procalcitonin concentrations provide useful biomarkers of infection severity and help guide the initiation and adjustment of antimicrobial regimens [14].

Beyond infection, non-infectious complications such as mucositis, hepatic dysfunction, or multi-organ failure can occur, particularly in frail or comorbid individuals. Prompt recognition and multidisciplinary management substantially improve outcomes.

## 4. Pathophysiology and Genetic Determinants

The pathophysiology of (DIA) is multifactorial, integrating immune, toxic, and genetic mechanisms that converge on profound myeloid suppression. Although these pathways vary among drug classes, they share final common outcomes involving neutrophil destruction and bone marrow progenitor depletion.

Several agents, particularly antithyroid drugs and antipsychotics, can induce drug-dependent antibodies that target circulating neutrophils or bone marrow precursors in the presence of the offending compound. These antibodies promote complement activation or opsonization, accelerating neutrophil destruction and causing maturation arrest within the marrow. The rapid and severe recurrence of agranulocytosis upon drug re-exposure strongly supports an immune-mediated mechanism.

Certain drugs, including clozapine, metamizole, and thionamides, generate reactive metabolites that exert direct cytotoxic effects on granulocytic progenitors. These intermediates can trigger oxidative stress, mitochondrial dysfunction, and apoptosis of myeloid precursors. The interplay between metabolic activation, detoxification capacity, and oxidative damage likely accounts for the substantial interindividual variability in susceptibility. Hepatic and myeloperoxidase-mediated bioactivation have been particularly implicated in clozapine-associated cases [15,16,17,18].

Genetic factors substantially modulate individual risk. Variants in HLA loci, notably HLA-B38, HLA-DRB1*04:02, and HLA-DQB1*03:02, have been reproducibly linked to clozapine- and methimazole-induced agranulocytosis across diverse populations [8]. Moreover, polymorphisms in drug-metabolizing enzymes, such as CYP2C19 and myeloperoxidase (MPO), may influence the generation and detoxification of reactive metabolites. These findings underscore the potential for pharmacogenomic screening to identify individuals at elevated risk, although widespread clinical application remains limited.

No single biomarker reliably predicts the risk, severity, or recovery trajectory of DIA. However, bone marrow cellularity and the presence of residual myeloid precursors have been correlated with faster hematologic recovery under granulocyte colony-stimulating factor (G-CSF) therapy, suggesting that marrow reserve and progenitor integrity are critical determinants of outcome. In select cases, bone marrow aspiration may offer a beneficial therapeutic alternative, particularly in the presence of thrombopenia/anaemia/pancytopenia. The selection of patients for this procedure should be restricted to those with a low risk of adverse drug reactions or an unknown risk profile [19]. Future research integrating genomic, proteomic, and immunologic profiling holds promise for refining risk stratification, guiding prophylaxis, and informing precision-based therapeutic strategies.

## 5. Comorbidities and Prognostic Factors

Several independent prognostic determinants of outcome in (DIA) have been consistently identified in multicentre studies [20,21]. Adverse prognosis is associated with the following factors:

- Age greater than 65 years;
- Renal impairment, whether acute or chronic;
- Bacteremia or septic shock at presentation;
- Delay between symptom onset and drug withdrawal;
- Multimorbidity, including diabetes mellitus, cardiovascular disease, or hepatic dysfunction.

In a large French multicentre retrospective cohort, mortality reached 14% among patients aged over 70 years presenting with renal dysfunction and bacteremia, compared to less than 3% in younger, otherwise healthy individuals [22]. Advanced age likely contributes to impaired bone marrow reserve, altered immune responses, and delayed neutrophil recovery, while comorbid conditions amplify infection-related morbidity.

These factors should be systematically assessed during diagnosis and integrated into clinical decision-making, particularly when evaluating the need for (G-CSF) therapy and intensive care support. Monitoring of blood counts for drugs known to cause neutropenia (e.g., clozapine), early recognition and withdrawal of the causative drug, combined with prompt antimicrobial treatment and hemodynamic stabilization, remain the cornerstone of management.

Beyond these classical determinants, emerging data suggest that genetic susceptibility, baseline inflammatory status, and marrow cellularity may further refine prognostic stratification, underscoring the need for prospective studies incorporating clinical, biologic, and genomic parameters.

## 6. Management Before G-CSF Introduction

Prior to the introduction of (G-CSF), the management of (DIA) relied primarily on supportive and anti-infective strategies. The cornerstone of treatment remains in the immediate withdrawal of the offending drug, which is the single most effective intervention to halt ongoing marrow suppression and immune activation.

All patients require hospital admission for close clinical observation and serial hematologic monitoring until (ANC) recovery. In febrile or clinically unstable individuals, empirical broad-spectrum antibiotic therapy must be initiated promptly, ideally within the first hour of fever detection, to cover both Gram-negative bacilli and *Staphylococcus aureus*. The choice of regimen typically mirrors that used in febrile neutropenia, including agents such as piperacillin-tazobactam, cefepime, or carbapenems, with or without vancomycin depending on local epidemiology and clinical context [21,23,24,25].

Corticosteroids are not routinely indicated, as their benefit in immune-mediated DIA remains unproven and their use may increase infection risk [26]. Prophylactic antifungal or antiviral agents are reserved for cases of prolonged neutropenia (>7–10 days), persistent fever despite broad-spectrum antibiotics, or profound immunosuppression.

Mortality in the pre-G-CSF era was largely determined by the speed of infection control and rapidity of neutrophil regeneration. Supportive care, including fluid resuscitation, oxygen therapy, and hemodynamic monitoring, remains essential to prevent septic complications. Early recognition of clinical deterioration and timely transfer to intensive care units for organ support can be lifesaving, particularly in elderly or comorbid patients.

The introduction of hematopoietic growth factors in the 1990s profoundly modified this landscape, enabling faster hematologic recovery and shorter hospitalization durations, a topic addressed in the following section.

## 7. Rationale for G-CSF Administration

G-CSF acts on bone marrow myeloid progenitors to promote proliferation, differentiation, and release of mature neutrophils into circulation. In DIA, where hematopoietic precursors are typically preserved, exogenous G-CSF accelerates neutrophil recovery and shortens the duration of severe neutropenia, thereby reducing exposure to infection risk.

Standard regimens employ filgrastim 5 μg/kg/day subcutaneously, continued until the (ANC) exceeds 1.0 × 10^9^/L for at least two consecutive days, with typical treatment durations of 3–7 days. Pegfilgrastim and lenograstim have been used in selected cases but lack robust comparative data in DIA [27].

### 7.1. Evidence of Efficacy

Evidence for G-CSF efficacy in DIA primarily derives from observational studies and meta-analyses. A meta-analysis of 13 studies involving 425 patients with antithyroid DIA demonstrated that G-CSF shortened the duration of neutropenia by a mean of 3.5 days (95% CI −4.5 to −2.5) compared with supportive care alone [28]. The Berlin Case–Control Study found that G-CSF administration reduced the median hospital stay by 50% (7 vs. 13 days) without significantly affecting mortality rates [1]. Similarly, a recent multicenter Spanish retrospective study (Gómez et al., 2023) including 168 patients confirmed faster ANC recovery with G-CSF (median 4.5 vs. 8.0 days, *p* < 0.001) and fewer severe infections, although mortality remained unchanged [29].

The benefit of G-CSF appears consistent across various drug etiologies:

- Clozapine-induced agranulocytosis: G-CSF or GM-CSF therapy halves recovery time (median 5 vs. 10 days) and may facilitate carefully supervised rechallenge in select psychiatric patients when no effective alternative exists [30].
- Metamizole-induced agranulocytosis: Recovery under G-CSF averages 3–5 days versus 7–10 days without stimulation, supporting its use in severe or septic presentations [31].

Across studies, G-CSF confers consistent clinical benefits, notably:

- Shorter duration of neutropenia (mean gain 3–5 days);
- Reduced length of hospitalization (mean reduction 2–4 days);
- Trend toward fewer infectious complications, though results vary by study.

However, a significant mortality reduction has not been consistently demonstrated. Mortality in modern cohorts is <5%, limiting statistical power to detect treatment differences. Furthermore, observational designs introduce potential confounding by indication, as G-CSF tends to be prescribed in the most severe cases, alongside improvements in overall supportive care over time [18,29].

### 7.2. Safety and Adverse Events

G-CSF is generally well tolerated in DIA. The most common adverse effects are mild bone pain, injection-site erythema, and transient fever. Rare but serious events include splenic enlargement, capillary leak syndrome, and acute lung injury, predominantly in septic or critically ill patients [32].

It is important to note that G-CSF does not increase the risk of relapses or trigger immune-mediated reactions and is considered safe in elderly patients and those with renal impairment. There is an absence of compelling evidence to suggest that short-term use in this context results in adverse hematologic or immunologic sequelae. However, an association has been observed between the prescription of prophylactic G-CSF and the subsequent development of AML or MDS (estimated cumulative incidence of 26 cases per 1000 patients) at 7 years in patients treated with G-CSF, compared with 14 cases per 1000 in the absence of treatment (estimated relative risk of 2.14) [33].

## 8. Determinants of G-CSF Use in Clinical Practice

The decision to initiate granulocyte colony-stimulating factor (G-CSF) therapy in (DIA) should be individualized, balancing disease severity, infection risk, and patient comorbidities (Table 2) [9]. Although most patients eventually recover following drug withdrawal, the timely use of G-CSF can accelerate neutrophil regeneration and mitigate infectious complications in high-risk groups.

Based on cumulative evidence from observational cohorts and expert consensus, G-CSF administration is recommended in the following clinical contexts:

- ANC < 0.1 × 10^9^/L, irrespective of infection status;
- ANC < 0.5 × 10^9^/L in the presence of fever, bacteremia, sepsis, or organ dysfunction;
- Elderly or multimorbid patients, particularly those with diabetes, cardiovascular disease, or renal impairment, who are at increased risk for infectious complications [9,19].

Conversely, in asymptomatic patients with ANC between 0.3 and 0.5 × 10^9^/L, no systemic infection, and favorable baseline health, spontaneous recovery is frequent following prompt discontinuation of the offending agent and supportive care alone.

Daily hematologic monitoring is advised until ANC normalization (>1.5 × 10^9^/L) to guide the need for continued or discontinued G-CSF therapy. This stratified approach optimizes the balance between efficacy, safety, and resource utilization while aligning with modern pharmacovigilance data.

Current evidence is limited by several factors:

- Absence of randomized controlled trials directly comparing G-CSF with placebo.
- Heterogeneity in drug classes, baseline patient severity, and dosing regimens.
- Publication bias favoring positive outcomes.
- Confounding by indication inherent in retrospective studies.

Future multicenter randomized trials are needed to determine the impact on mortality, assess cost-effectiveness, and establish optimal dosing strategies, including pegylated formulations.

**Figure 1:**
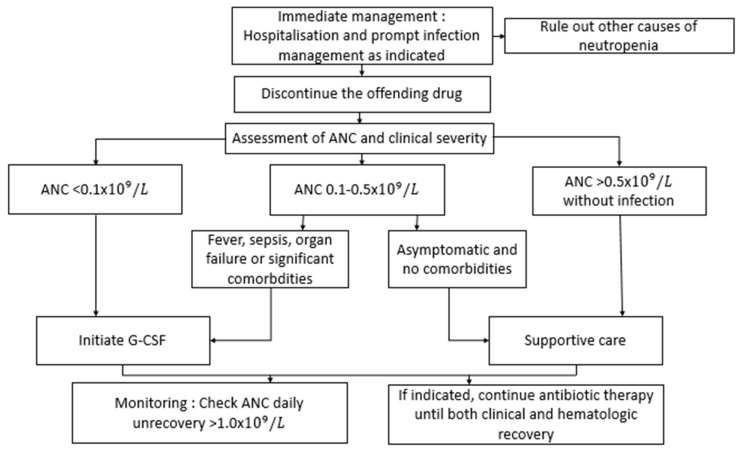
Practical algorithm for management of drug-induced neutropenia and granulocyte colony-stimulating factor (G-CSF) therapy. ANC: Absolute Neutrophil count.

Figure 1 Propose a practical algorithm for management of drug-induced neutropenia and G-CSF therapy.

It is important to report cases of DIA to the responsible pharmacovigilance authorities as this is a vital step that can only be performed by the clinicians who deal with these patients.

## 9. Conclusions

Drug-induced agranulocytosis remains a rare but potentially life-threatening adverse drug reaction. G-CSF consistently shortens the duration of neutropenia and hospitalization, with a probable reduction in infectious complications, although a definitive mortality benefit has not been established. Decisions regarding G-CSF use should be guided by clinical severity, comorbidities, and neutrophil count, supporting a pragmatic, risk-adapted approach until robust prospective evidence is available.

This study is based upon decades of research. However, it should be noted that there are no large-scale prospective randomized studies specifically dedicated to the analysis of agranulocytosis. This phenomenon can be partially attributed to the logistical challenges associated with anticipating such events, as well as to considerations of ethical principles. Indeed, it appears essential to expeditiously treat these patients. Moreover, when G-CSF administration is deemed appropriate, a comparison between groups receiving G-CSF and those receiving a placebo becomes challenging.

## 10. Future Directions

Future research priorities include the establishment of prospective registries with standardized data collection, pharmacogenetic profiling to identify susceptible individuals, and health–economic analyses assessing cost-effectiveness and impact on quality of life. Integration of these parameters into a predictive risk score could facilitate personalized therapeutic strategies, optimize resource allocation, and improve patient outcomes.

## Figures and Tables

**Table 1:** Major epidemiologic and clinical determinants of drug-induced agranulocytosis.

Determinant	Description/Association	Clinical Impact
Age > 65 years	Higher susceptibility and mortality	Increased risk of sepsis and prolonged recovery
Female sex	Predominance in several cohorts (antithyroid, antibiotic exposure)	Reflects exposure pattern
Causative drug	Antithyroid, clozapine, metamizole, β-lactams, cotrimoxazole	Determines latency and recovery profile
Time to onset	Usually 2–12 weeks after exposure	Early detection improves outcome
ANC < 0.1 × 10^9^/L	Predicts severe infection and death	Strong indication for G-CSF
Renal dysfunction	Independent predictor of mortality	Requires early intervention
Bacteremia/sepsis	Occurs in 40–60%	Major determinant of mortality
Delayed drug withdrawal	Worsens prognosis	Early cessation essential
Comorbidities (diabetes, CV disease)	Reduce immune reserve	Increase infection risk

**Table 2:** Practical determinants of granulocyte colony-stimulating factor (G-CSF) use in drug-induced agranulocytosis.

Determinant	Criteria/Considerations	Evidence/Notes	Impact on G-CSF Use
Neutrophil count	Profound neutropenia (<0.1 × 10^9^/L)	Observational cohorts and meta-analyses show faster recovery with G-CSF in severe neutropenia	Strong driver for initiation
Infection/sepsis severity	Severe infection, septicemia, hemodynamic instability	Faster neutrophil recovery associated with reduced infectious complications in high-risk patients	High priority for G-CSF
Age	Older adults (>65 years)	Age-related immune senescence increases risk of complications	Supports earlier or more aggressive G-CSF use
Comorbidities	Cardiac, renal, hepatic disease	Comorbidities linked to prolonged hospitalization and infection risk	Favors G-CSF in high-risk patients
Causative drug/etiology	Clozapine, antithyroid drugs, metamizole	Some drugs associated with more severe or prolonged agranulocytosis	Influences clinician decision toward G-CSF
Clinical course	Slow spontaneous recovery, prolonged neutropenia	Case series indicate delayed recovery without G-CSF may increase complications	Supports initiation if delayed recovery observed
Institutional protocol/experience	Local guidelines, physician familiarity	Lack of RCTs leads to empiric, risk-adapted strategies	Variability exists; guideline-driven use preferred

## Data Availability

The data presented in this study are available on request from the corresponding author. The data are not publicly available due to privacy restrictions.

## Abbreviations

The following abbreviations are used in this manuscript:

DIA	Drug-induced agranulocytosis
ANC	Absolute Neutrophil count
GCSF	Granulocyte colony-stimulating factor
NSAIDs	nonsteroidal anti-inflammatory drugs
CV	Cardio-vascular
CRP	C-reactive protein
MPO	myeloperoxidase
STAT	signal transducers activators of transcription
